# Prehospital prediction of hospital admission for emergent acuity patients transported by paramedics: A population-based cohort study using machine learning

**DOI:** 10.1371/journal.pone.0289429

**Published:** 2023-08-24

**Authors:** Ryan P. Strum, Fabrice I. Mowbray, Manaf Zargoush, Aaron P. Jones

**Affiliations:** 1 Department of Health Research Methods, Evidence and Impact, McMaster University, Hamilton, Ontario, Canada; 2 College of Nursing, Michigan State University, East Lansing, Michigan, United States of America; 3 Department of Health Policy and Management, McMaster University, Hamilton, Ontario, Canada; 4 Institute for Clinical Evaluative Sciences, McMaster University, Hamilton, Ontario, Canada; Drexel University, UNITED STATES

## Abstract

**Introduction:**

The closest emergency department (ED) may not always be the optimal hospital for certain stable high acuity patients if further distanced ED’s can provide specialized care or are less overcrowded. Machine learning (ML) predictions may support paramedic decision-making to transport a subgroup of emergent patients to a more suitable, albeit more distanced, ED if hospital admission is unlikely. We examined whether characteristics known to paramedics in the prehospital setting were predictive of hospital admission in emergent acuity patients.

**Materials and methods:**

We conducted a population-level cohort study using four ML algorithms to analyze ED visits of the National Ambulatory Care Reporting System from January 1, 2018 to December 31, 2019 in Ontario, Canada. We included all adult patients (≥18 years) transported to the ED by paramedics with an emergent Canadian Triage Acuity Scale score. We included eight characteristic classes as model predictors that are recorded at ED triage. All ML algorithms were trained and assessed using 10-fold cross-validation to predict hospital admission from the ED. Predictive model performance was determined using the area under curve (AUC) with 95% confidence intervals and probabilistic accuracy using the Brier Scaled score. Variable importance scores were computed to determine the top 10 predictors of hospital admission.

**Results:**

All machine learning algorithms demonstrated acceptable accuracy in predicting hospital admission (AUC 0.77–0.78, Brier Scaled 0.22–0.24). The characteristics most predictive of admission were age between 65 to 105 years, referral source from a residential care facility, presenting with a respiratory complaint, and receiving home care.

**Discussion:**

Hospital admission was accurately predicted based on patient characteristics known prehospital to paramedics prior to arrival. Our results support consideration of policy modification to permit certain emergent acuity patients to be transported to a further distanced ED. Additionally, this study demonstrates the utility of ML in paramedic and prehospital research.

## Introduction

Canadian paramedics utilize the Canadian Triage and Acuity Scale (CTAS) to classify medical acuity and patient care needs in the prehospital setting [1, 2]. Health policy mandates paramedics transport patients with an emergent acuity CTAS score to the closest emergency department (ED) for time-sensitive emergency medicine [1]. However, the majority of emergent acuity patients do not receive emergency medicine, experience clinically deteriorate or need hospital admission (56.6%); instead, a significant proportion receive physician care resembling primary care (39.3%) [3–5]. The exclusive transport of all emergent acuity patients to the closest ED represents an operational inefficiency. All emergent acuity patients are handled similarly in the prehospital field with transport to the closest ED, regardless of patient repatriation consideration for continuity of care, patient preference, ED workload, or bed availability at further distanced hospitals [1]. In circumstances where emergent acuity patients do not require hospital admission or time-sensitive care, transport to a further distanced ED could beneficial for both the patient and Eds [6]. Such an approach could be similar to the handling of urgent and low acuity patients who may be transported to any of the three nearest hospitals [6].

Knowing which prehospital patient characteristics predict ED admission could inform policy modifications and guide paramedics to transport emergent acuity patients to the closest ED, rather than consider further distanced EDs. Utilization of machine learning (ML) classifications algorithms could identify patient characteristics that are predictive of hospital admission among emergent acuity patients, thereby aiding in the determination of which patients are unsuitable for bypassing the closest ED. ML has the potential to improve care efficiency in patient distribution, and support paramedic decision-making when clinical information is limited in the prehospital field [7, 8].

Our primary objective was to utilize ML algorithms to predict hospital admission for adult patients arriving at the ED through paramedic transport with an emergent acuity triage score. Our secondary objective was to evaluate the predictive utility of patient characteristics known to paramedics in the prehospital setting as predictors of ED outcomes in ML.

## Material and methods

### Study design

We conducted a population-level cohort study using administrative ED patient records to analyze model performance of four machine learning algorithms. Patient medical records completed by paramedics in the prehospital field are not available for research purposes in Ontario; we approximated characteristics that would be known to paramedics prior to hospital arrival by using ED data collected upon entry to the department at nurse triage. The STROBE statement was followed for the reporting of results (S1 Table) [9].

### Population

All Ontario adult patients (≥18 years) transported to the ED by ground paramedics after use of the emergency alert system (9-1-1) were eligible for inclusion. We included high acuity patients with an ‘emergent’ CTAS triage score (two). High acuity patients are classified as having a CTAS score of one and two, of which patients with a CTAS one score could not be considered for any plausible hospital bypass due to their life-threatening medical severity, excluding them. CTAS is an ordinal scale that ranges from one to five, with a score of one indicating the highest acuity (resuscitation) and five the lowest acuity (non-urgent) [2]. We excluded ED visits transported by air paramedic medical services, and visits with an outcome other than hospital admission or ED discharge due to data availability. All ED visits between January 1, 2018 to December 31, 2019 that met the inclusion criteria were included, as this timeframe represents the most recently available two-year period prior to the Covid-19 pandemic when paramedic and ED utilization trends changed [10].

### Data source

We used population-level administrative ED patient data from the National Ambulatory Care Reporting System (NACRS) database. NACRS is a hospital and community-based ambulatory care administrative database that collects patient and visit-related data at ED triage and the time care is provided [11, 12]. All Ontario hospitals are required to submit electronic patient abstracts from the ED to comply with reporting and quality measures and these data are recorded in NACRS. NACRS data was accessed through the ICES (formerly known as the Institute for Clinical Evaluative Sciences); ICES is a non-profit, independent corporation that supports the study of health service and population-wide outcomes in Ontario. All data were provided in November 2021, and accessed through ICES’s secure server IDAVE.

### Ethics

ICES’ collection and use of NACRS secondary ambulatory data are authorized under Section 45 of Ontario’s Personal Health Information Protection Act (PHIPA) as a prescribed entity, which is exempt from review by a Research Ethics Board [13, 14]. The use of the data in this study is authorized and approved by ICES’ Privacy and Legal Office, which does not require participant consent according to national regulations in Ontario, Canada [13]. All data were anonymized prior to access for this research.

### Predictor eligibility and outcome variables

To be pragmatic, we extracted all patient characteristics collected at ED triage from the NACRS database for eligibility in the study. These characteristics constitute a comprehensive representation of patients, their conditions, rationale for visit prior to ED arrival, and would be known to paramedics in the prehospital setting (paramedics routinely provide this data to ED triage). We used hospital admission from the ED as the outcome, defined as a binary variable where a patient is hospitalized to receive further clinical treatment and assessment [15].

### Predictor selection and data preparation

We selected eight characteristic classes for inclusion in the study based on data availability and completeness in NACRS, previous literature and clinical importance determined *a priori* by emergency physicians at our local academic hospital centre. Selected characteristic classes were termed the predictors in the study. Missing data for each included characteristic were scant (≤0.5%), and directly stated where applicable; complete case analysis was utilized. We dummy-coded categorical predictor variables for non-tree-based algorithm models [16].

All predictors were measured and recorded upon entry to the ED at triage, and prior to any ED diagnostic tools being employed. Patient age was originally extracted as twenty-two categorical levels due to personal health information privacy restrictions, and further collapsed into three categories to parallel major age progressions: 18–39, 40–64, 65–105 years. Access to primary healthcare is defined as having access to any of a family physician, family health team, walk-in clinic or other primary care setting. Geographic location of the ED is classified as either urban or rural; considered urban when the community (defined by regional postal codes) are equal to or greater than 10,000 residents. The presenting complaint refers to the primary reason for seeking emergency medical care, categorized using the Canadian Emergency Department Information System (CEDIS). Referral source indicates the entity that advised the patient to seek emergency care in the ED.

### Statistical analysis and machine learning algorithms

We reported descriptive statistics using measures of central tendency and frequency. We trained four independent ML classification algorithms in this study: logistic regression (LR), lasso logistic regression (Lasso LR), random forest (RF) and gradient boosted trees (GBT). We selected algorithms based on previous literature to predict patient ED visit outcomes in consultation with health care machine learning experts [17–19]. We used 10-fold cross-validation to calculate predictive performance for the models using the same series of predictors. Cross-validation is a reliable measure of model performance and an approach commonly utilized in machine learning health care prediction research [20]. We used a dichotomous threshold of 50% likelihood for our predictions. We managed and analyzed the data in R software, V.3.6 and built the machine learning algorithms using ‘*mlr’* and ‘*mlr3’* packages [21, 22]. The R script that defined the study cohort is shown in S1 File; the R script that computed the models could not be extracted due to ICES privacy restrictions.

### Hyperparameter tuning

We utilized hyperparameter optimization in RF and GBT algorithms to maximize the average 10-fold cross validation area under the receiver operating curve (AUC) through tuning prior to computation of the final models [23]. For the RF algorithm, we used an exhaustive grid search for eligible tuning parameters and further trained models by optimizing the minimum node size and number of splitting variables. For GBT, we employed a broad random search of parameter space, followed by a focused random search within the best performing areas of the broad search. The hyperparameters trained in the GBT algorithm consisted of maximum number of iterations, maximum tree depth, shrinkage rate, minimum sum of instance weights required to stop splitting and gamma. A comprehensive depiction of all hyperparameter tuning used for the RF and GBT algorithms are shown in the S2 Table.

### Performance measurement and predictor importance

We measured the performance of all algorithms using the AUC that resulted from 10-fold cross-validation with corresponding 95% confidence intervals (CIs). We assessed calibration accuracy using the Brier Scaled score, a measure of probabilistic predictions on a scale of 0 (perfect accuracy) to 1 (perfect inaccuracy) [24]. Additionally, we reported the sensitivity, specificity, positive predictive value, and negative predicted value for each algorithm. To determine the importance of each predictor, we used permutation methods to calculate the average decrease in AUC in the absence of a predictor. The predictors with the highest variable importance corresponded to the largest declines in AUC. To construct a comprehensive list of variable importance across all algorithms, we computed the mean reduction in AUC for all predictors by averaging their AUC reduction from all models. We ranked the mean AUC reductions to produce a top 10 most important predictors list.

## Results

### Descriptive results

**Fig 1:**
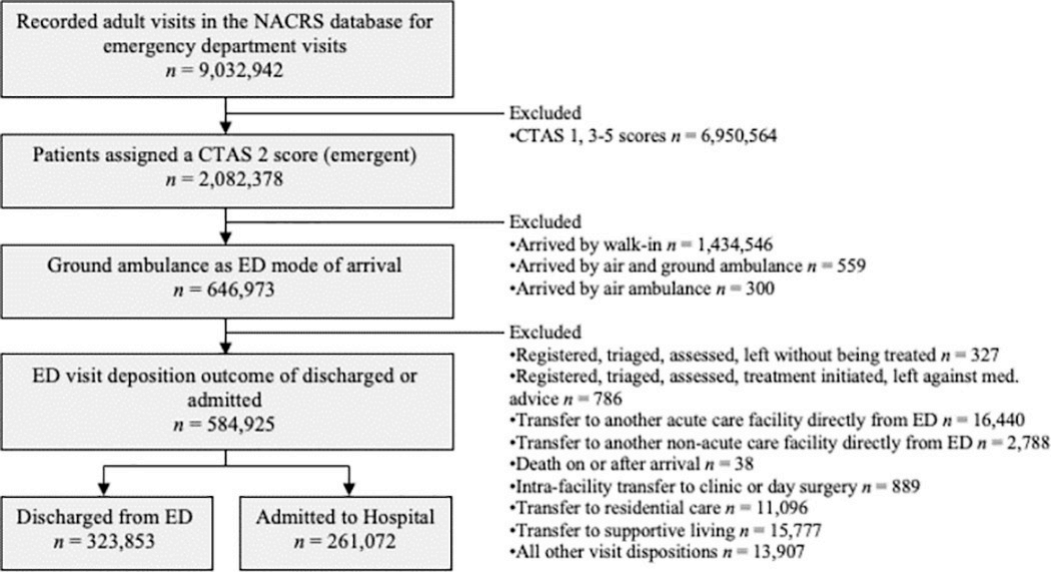
Flow diagram of patient inclusion for study of emergency department visits transported by paramedics and assigned an emergent triage acuity score.

Fig 1 shows exclusions used to construct the study cohort. A total of 584,925 ED patients transported by paramedics were included, yielding 261,072 (44.6%) patients admitted to hospital and 323,853 (55.4%) patients discharged directly from ED. Overall, our cohort represented 90.4% of all adult emergently triaged paramedic transported ED visits in Ontario.

Table 1 shows a summary of the clinical profile of all patients in our study cohort. The majority of patients were older adults aged 65 years or greater (296,111; 50.6%), male (296,902; 50.8%), referred to ED by self of family (490,242; 83.3%), previously diagnosed with hypertension (336,174; 57.5%) and had their ED visit occur in an urban region of Ontario (535,270; 91.5%). The most common presenting complaints on ED arrival were cardiovascular (178,524; 30.5%), neurologic (99,119; 16.9%) and gastrointestinal (61,104; 10.4%). Missing data was negligible and non-informative, presenting only in the geographic location of the ED (0.5%).

**Table 1:** Prehospital characteristics of emergent acuity emergency department patients transported by paramedics in Ontario, Canada, January 1, 2018 to December 31, 2019.

Patient Characteristic	N (%)
**Age Group**, years	
18–39	112,260 (19.2)
40–64	176,554 (30.2)
65–105	296,111 (50.6)
**Sex**	
Male	296,902 (50.8)
Female	288,023 (49.2)
**Comorbidities** ^a^	
Hypertension	336,174 (57.5)
Diabetes	183,657 (31.4)
Chronic Obstructive Pulmonary Disease	160,216 (27.4)
Asthma	133,618 (22.8)
Rheumatoid Arthritis	16,279 (2.8)
Congestive Heart Failure	105,367 (18.0)
Bowel Disease	7,747 (1.3)
Cancer	98,377 (16.8)
**Access to Primary Healthcare** ^b^	
Yes	500,373 (85.5)
No	84,552 (14.5)
**Presenting Complaint** ^c^	
Cardiac Arrest	125 (0.0)
Cardiovascular	178,523 (30.5)
ENT (ears, nose, throat)	10,260 (1.8)
Environmental	1,488 (0.3)
Gastrointestinal	61,104 (10.4)
Genitourinary	10,913 (1.9)
Mental Health	27,382 (4.7)
Neurologic	99,119 (16.9)
Obstetrician-Gynecological	2,805 (0.5)
Ophthalmology	1,565 (0.3)
Orthopedic	37,516 (6.4)
Respiratory	70,078 (12.0)
Skin	5,644 (1.0)
Substance Misuse	32,459 (5.5)
Trauma	9,297 (1.6)
General/Minor	32,206 (5.5)
Other	4,441 (0.8)
**Geographic Location of ED** ^d^	
Urban	535,270 (91.5)
Rural	46,771 (8.0)
Missing	2,884 (0.5)
**Referral Source**	
Self/Family Member	490,242 (83.8)
Ambulatory Care Service	7,169 (1.2)
Private Practice	4,762 (0.8)
Residential Care Facility	44,706 (7.6)
Other	38,046 (6.5)
**Receiving Home Care Services**	
Yes	129,628 (22.2)
No	455,297 (77.8)
**ED Visit Disposition Outcome**	
Discharged	323,853 (55.4)
Admitted	261,072 (44.6)

Note: ED = emergency department.

^a^ Conditions present on ED arrival.

^b^ Identifies if a patient has access to either a family physician, family health team, walk-in clinic or other primary care setting.

^c^ Categorized by the Canadian Emergency Department Information System (CEDIS) of the Canadian Institute for Health Information (CIHI).

^d^ Determined using forward sortation area (FSA) of Ontario postal codes.

### Hyperparameter tuning and comparison of predictive model performance

Table 2 displays the final predictive model performance of all machine learning algorithms used in this study after benchmarking. Overall, each algorithm performed similarly with an AUC ranging from 0.77–0.78. The GBT model computed the best overall performance in classifying paramedic transported emergently triaged visits that were admitted to hospital, achieving an AUC of 0.78 (95% CI 0.78–0.78). The LR and lasso LR models computed the most accurate predictions with a Brier Scaled score of 0.22. S2 Table shows the details of hyperparameter tuning ranges and optimal values used to produce the final RF and GBT algorithm outputs. Calibration plots of predicted probabilities were assessed for quality of algorithm predictions; all algorithms received excellent calibration with slopes ranging from 0.997 to 1.086, shown in S1–S4 Figs.

**Table 2:** Measures of predictive accuracy in four machine learning algorithms.

Measure	LR	Lasso LR	RF	GBT
**AUC (95% CI)**	0.77 (0.76–0.77)	0.77 (0.76–0.77)	0.78 (0.77–0.78)	0.78 (0.78–0.78)
**Brier Scale**	0.22	0.22	0.24	0.24
**Sensitivity**	0.80	0.81	0.76	0.78
**Specificity**	0.58	0.57	0.63	0.37
**PPV**	0.70	0.70	0.71	0.71
**NPV**	0.71	0.71	0.70	0.71

**Note**: LR = logistic regression, Lasso LR = lasso logistic regression, RF = random forest, GBT = gradient boosted trees, AUC = area under the receiver operating characteristic curve, CI = confidence intervals, PPV = positive predictive value, NPV = negative predictive value.

### Variable importance

**Fig 2:**
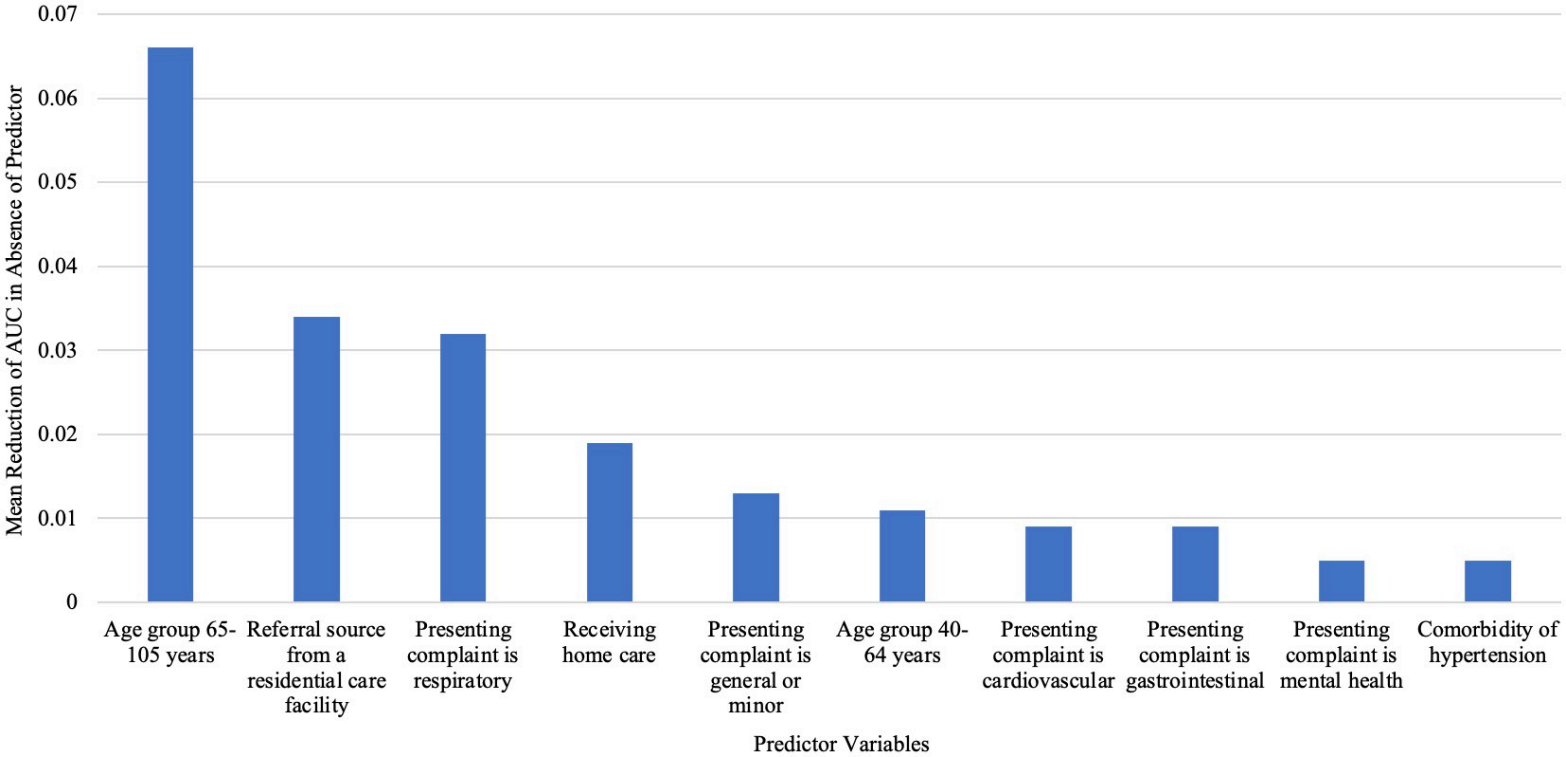
Mean relative importance for the top 10 patient characteristic predictors of hospital admission across all machine learning algorithms.

Fig 2 shows the mean variable importance score for the top 10 predictors of hospital admission from the ED following paramedic transport. The top 10 most important predictors were (1) age 65 to 105 years, (2) referred to ED from a residential care facility, (3) presenting complaint is respiratory, (4) receiving home care, (5) presenting complaint is general or minor, (6) aged 40 to 64 years, (7) presenting complaint is cardiovascular, (8) presenting complaint is gastrointestinal, (9) presenting complaint is mental health and (10) comorbidity of hypertension. S3 Table shows the relative importance of each predictor across all machine learning algorithms, presented as the reduction in AUC in absence of the predictor. S4 Table shows the variable-relevance ranking of the top 10 predictors for each ML algorithm. Aged 65 to 105 years was consistently the most important variable ranked amongst all ML algorithms. Referral from residential care was ranked as either the second or third most important predictor, and presenting with a respiratory complaint was ranked between second to fourth position. Receiving home care’s rank was spread from second to sixth in the algorithms. All remaining top 10 predictors were distributed variably in each algorithm, with less uniformity in rankings.

## Discussion

We predicted hospital admission using readily available clinical characteristics (age, gender, presenting complaint, comorbidities) and non-clinical characteristics (geographic location of ED, referral source, receiving home care services) known to paramedics prior to ED arrival. The acceptable algorithm results demonstrated that prehospital characteristics have predictive validity to forecast ED visit dispositions of emergent acuity. In the absence of additional important clinical and non-clinical characteristics not captured in administrative data, we computed acceptable AUC outputs of 0.77–0.78 and probabilistic accuracy scores of 0.22–0.24 across all algorithms. All machine learning algorithms performed similarly in all measures of statistical precision (sensitivity, specificity).

Our study identified older age, living in a residential facility, presenting to ED with a respiratory complaint, and receiving home care as the most informative predictors of hospital admission. These findings align with previous literature that examined predictors of admission for unscheduled ED visits [25–28]. A plethora of research has examined patient characteristics predictive of admission in the older adult cohorts, yielding consistent results with our predictors [29–32]. Receiving home care was included less frequently as an admission predictor in the literature, suggesting a potential underreporting of this characteristics influence on ED visit dispositions. Our results highlight that later-stages of life and requiring support care (residential or institutional) are the driving forces of ED admission from prehospital patient characteristics.

Several characteristics were non-informative in predicting hospital admissions, such as ED location, presenting complaints related to environmental reasons or the skin, comorbidities of bowel disease or asthma, and referral from ambulatory care services or private practices. These non-informative predictors align somewhat with prior work [4, 33]. Specifically, comorbidities of asthma and diabetes have been reported as risk markers for hospital admission, although not consistently in the literature [34, 35]. In our study, these two predictors did not contribute significantly to the prediction of admission. This result may be attributed to the inclusion of only paramedic transported visits, as paramedics frequently treat asthma or diabetes conditions prior to ED arrival in the prehospital field, thereby decreasing the likelihood of subsequent admission.

Considering the similarities in discriminative and calibration accuracy between the LR model and more advanced ML algorithms, it is worth noting that this model may be best suited for pragmatic implementation and decision-making at the clinical and policy levels. Interpretation of relative odds and confidence intervals are standard in medical education, whereas ML interpretation is not. The simplicity of the LR supports an easier comprehension of the study findings by medical and health policy professionals, who are most likely to influence policy changes.

Our study holds relevance for the scientific community as it employs ML algorithms to predict ED outcomes of paramedic transported patients using only characteristics known before hospital arrival. Given these predictors in the study are easily accessed by paramedics in the prehospital setting, policymakers could consider modifications to patient distribution legislation and clinical decision rules to transport certain patients to further distanced EDs. Our study’s most important predictors could identify ineligible patients for transport to a further distanced hospital due to their increased likelihood for admission.

One limitation of our study was the absence of additional clinical and non-clinical characteristics available to paramedics, which could improve prediction performance. Our data was limited to translating ED data collected at triage to what would be known in the prehospital field. This pragmatic approach was necessary due to the challenges associated with accessing paramedic administrative data resources, which are held independently by each of the 52 paramedic service independently and do not regularly permit research access [8]. Some degree of heterogeneity may exist between prehospital and ED clinicians categorizing the primary complaint, although we hypothesize that this is minimal given the broad categorizations of the conditions. Additionally, we used ED disposition as the outcome of our primary objective as a proxy for poor clinical condition and the need for further treatment due to data availability. Alternative strategies, such as emergency procedures or administration of emergency medicine, could provide more precise indicators of poor clinical condition, but are not routinely available for research.

With advanced knowledge of which patient characteristics are most predictive of admission, a paramedic distribution protocol could be developed to stratify emergent patients into a two-tier system. The first group could comprise patients who must be transported to the closest ED, as they have characteristics associated with a high predicted probability of admission. The second group could consist of patients eligible for transport to the most appropriate hospital, even if further distanced, as their characteristics are not associated with an increased predicted probability of admission. Future research is needed to determine the inclusion criteria of these groups within emergently triaged patients. Nevertheless, our study serves as a first step towards investigating the concept of hospital redistribution for high acuity patients. Employing threshold tuning to identify an ideal cut-point in the predicted probability with an acceptable sensitivity could be beneficial in defining these groups. Stratification of patients assigned an emergent triage score into groups could optimize patient distribution, improve ED congestion and plausibly improve patient-important outcomes. However, integration of our results should not be readily translated into the clinical setting without external validation or examining temporal validation data.

An overarching goal of this study was to determine if patient characteristics known prehospital could predict ED visit dispositions prior to arrival. Our study demonstrated that prehospital characteristics have predictive utility for forthcoming ED visits. We established that ML has ample potential for integration into prehospital research and could be an important methodology for optimizing paramedic clinical practices and logistical effectiveness. Including paramedic medical reports, linked to ED administrative data resources, would undoubtedly increase the probabilistic accuracy of predictive algorithms, and contribute variables that encompass a more complete prehospital patient presentation than ED resources alone.

Future research should leverage ML to analyze the utility of patient characteristics in predicting ED visit dispositions in low acuity transports. Ontario paramedics could be granted the capacity to transport low acuity patients to non-ED community-based care alternatives to ease ED patient congestion, though identifying and classifying of suitable patients for redirection from an ED poses challenging in the community setting [36]. Future research on low acuity transports should utilize ML to investigate which patient characteristics are most predictive of ED visits where patients are discharged and did not require any the emergency medicine intervention [36]. Knowledge of these predictors would be beneficial in supporting the development of paramedic clinical guidelines to identify patients potentially suitable for redirection, considering the limited clinical information available to paramedics. Additionally, future research could replicate our results using emergency medicine procedures as the primary outcome to provide a plausibly more precise depiction of patients with poor clinical conditions in the ED. Lastly, external validity research using multijurisdictional data outside of Ontario could further examine our results for clinical implementation purposes.

## Conclusion

Hospital admission was predicted with acceptable accuracy using patient characteristics of paramedic transported patients with an emergent acuity. All ML algorithm performed similarly with an acceptable AUC and probabilistic accuracy score. Our results could support the exploration of policy modification to consider the transport of certain emergently triaged patients to a further distanced EDs when appropriate. Our study highlights the abundant predictive utility of prehospital patient characteristics in ML, emphasizing its potential in future paramedic and prehospital research.

## Supporting information

S1 File R script. R script to define the study cohort from raw data.
(DOCX)

S1 Fig Calibration of logistic regression. (TIF)

S2 Fig Calibration of lasso logistic regression. (TIF)

S3 Fig Calibration of random forest. (TIF)

S4 Fig Calibration of gradient boosted trees. (TIF)

S1 Table STROBE statement. Checklist of items that should be included in reports of *cohort studies*.
(DOCX)

S2 Table Hyperparameter tuning details. (DOCX)

S3 Table Variable importance. Results of variable importance predictors for each machine learning algorithm.
(DOCX)

S4 Table Variable-relevance ranking. Variable-relevance ranking of top 10 predictors for each machine learning algorithm.
(DOCX)
