# Supplementary material for: Prehospital prediction of hospital admission for emergent acuity patients transported by paramedics: A population-based cohort study using machine learning
Source: PLoS One. 2023 Aug 24;18(8):e0289429. doi: 10.1371/journal.pone.0289429 (PMC10449470; doi:10.1371/journal.pone.0289429)
Supplement: S1 File — R script to define the study cohort from raw data. (DOCX) [file pone.0289429.s001.docx]

**S1 File. R Script.** R script to define the study cohort from raw data.

Library(dplyr)

Library(mlr3)

Library(ggplot2)

#study cohort

alldata = read.csv(file.choose())

emerg_data = alldata %>% filter(triage == “2”)

emerg_data = emerge_data %>% filter(admambul == “G” | admambul == “N”)

emerg_data = emerg_data %>% filter(urgentcare == 0)

#selection of study variables

cohort_data = select(emerg_data, age_group, sex, accessphc, hyper, diabetes, copd, asthma, RA, CHF, bowel_disease, cancer, admambul, complaint, rural, refersource, visdisp2005, HCD)

cohort_data = cohort_data %>% filter(visdisp2005 == 1 | visdisp2005 == 6 | visdisp2005 == 7 | visdisp2005 == 15 | visdisp2005 == 16 | visdisp2005 == 17)

cohort_data = cohort_data %>% mutate(outcome = ifelse(visdisp2005 == 6 | visdisp2005 == 7, “0”, “1”))

cohort_data = cohort_data %>% mutate(age = ifelse(age_group == “40-44” | age_group == “45-49” | age_group == “50-54” | age_group == “55-59” | age_group == “60-64”, “40-64”, ifelse(age_group == “65-69” | age_group == “70-74” | age_group == “75-79” | age_group == “80-84” | age_group == “85-89” | age_group == “90-94” | age_group == “95-99” | age_group == “100-105”, “65-105”, “18-39”))))

cohort_data = rename(cohort_data, transportmode = admambul)

cohort_data = cohort_data %>% mutate(refer = ifelse(refersource == 1, “self”, ifelse(refersource == 3, “ambulatory care service”, ifelse(refersource == 4, “private practice”, ifelse(refersource == 7, “residential care facility”, “other”))))

#conversion to factors for machine learning

ml_data = select(cohort_data, outcome, age, sex, accessphc, hyper, diabetes, copd, asthma, RA, CHF, bowel_disease, cancer, transportmode, complaint, rural, refer, homecare)

ml_data$age = as.factor(ml_data$age)

ml_data$acsc = as.factor(ml_data$acsc)

ml_data$refer = as.factor(ml_data$refer)
