# Supplementary figures and images for: Prehospital prediction of hospital admission for emergent acuity patients transported by paramedics: A population-based cohort study using machine learning
Source: PLoS One. 2023 Aug 24;18(8):e0289429. doi: 10.1371/journal.pone.0289429 (PMC10449470; doi:10.1371/journal.pone.0289429)

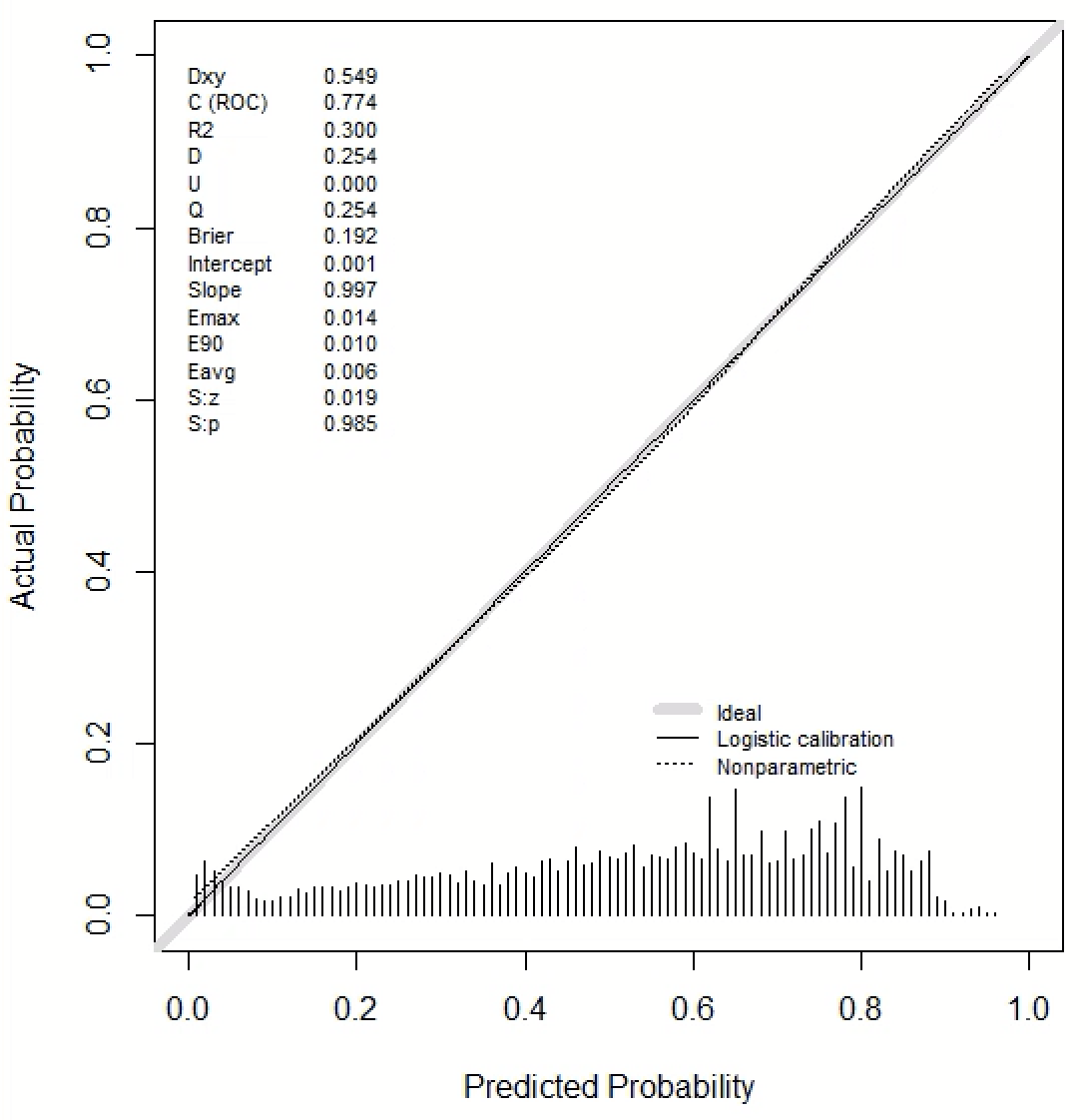

Supplement: S1 Fig — (TIF) [file pone.0289429.s002.tif]

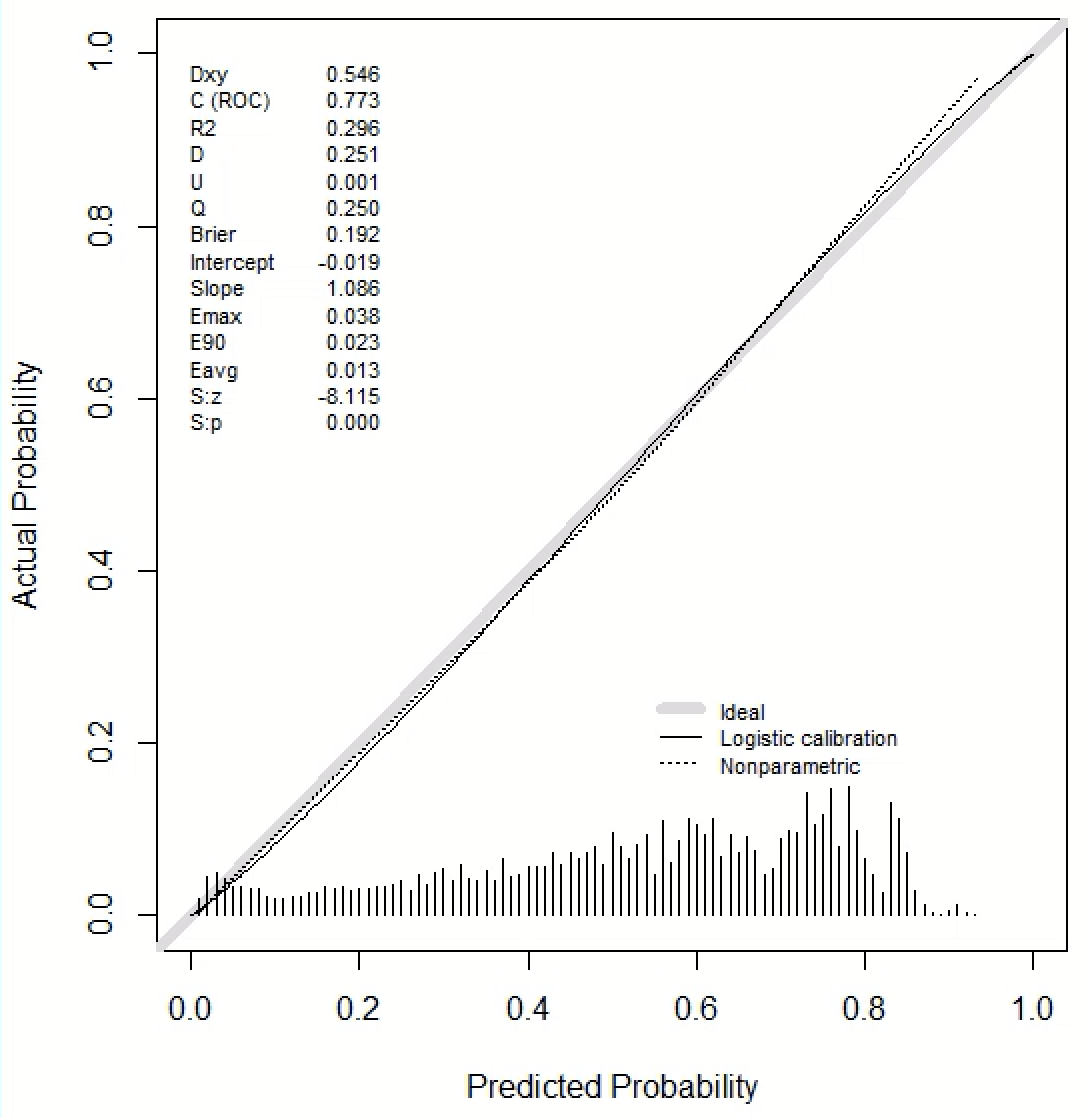

Supplement: S2 Fig — (TIF) [file pone.0289429.s003.tif]

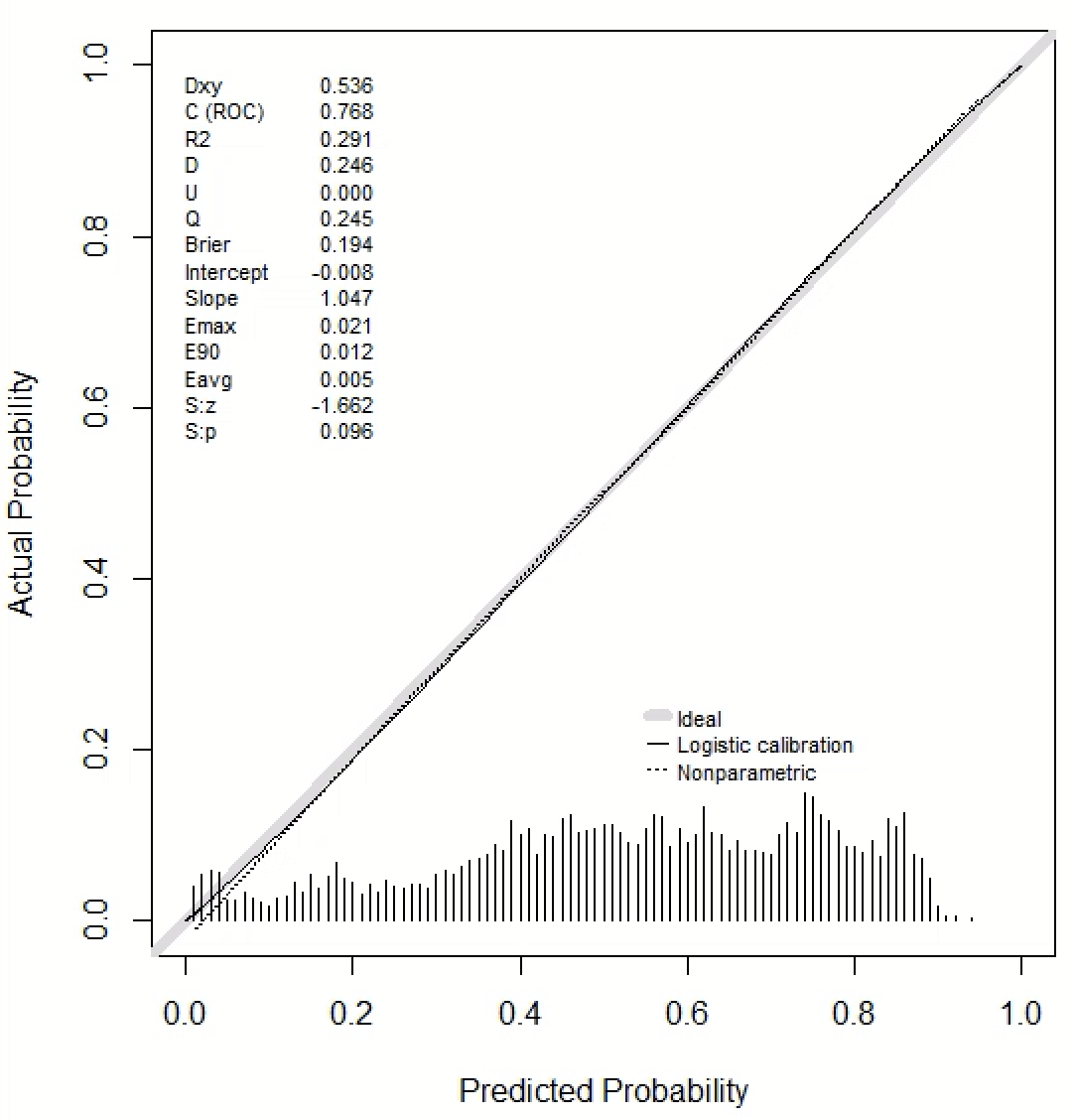

Supplement: S3 Fig — (TIF) [file pone.0289429.s004.tif]

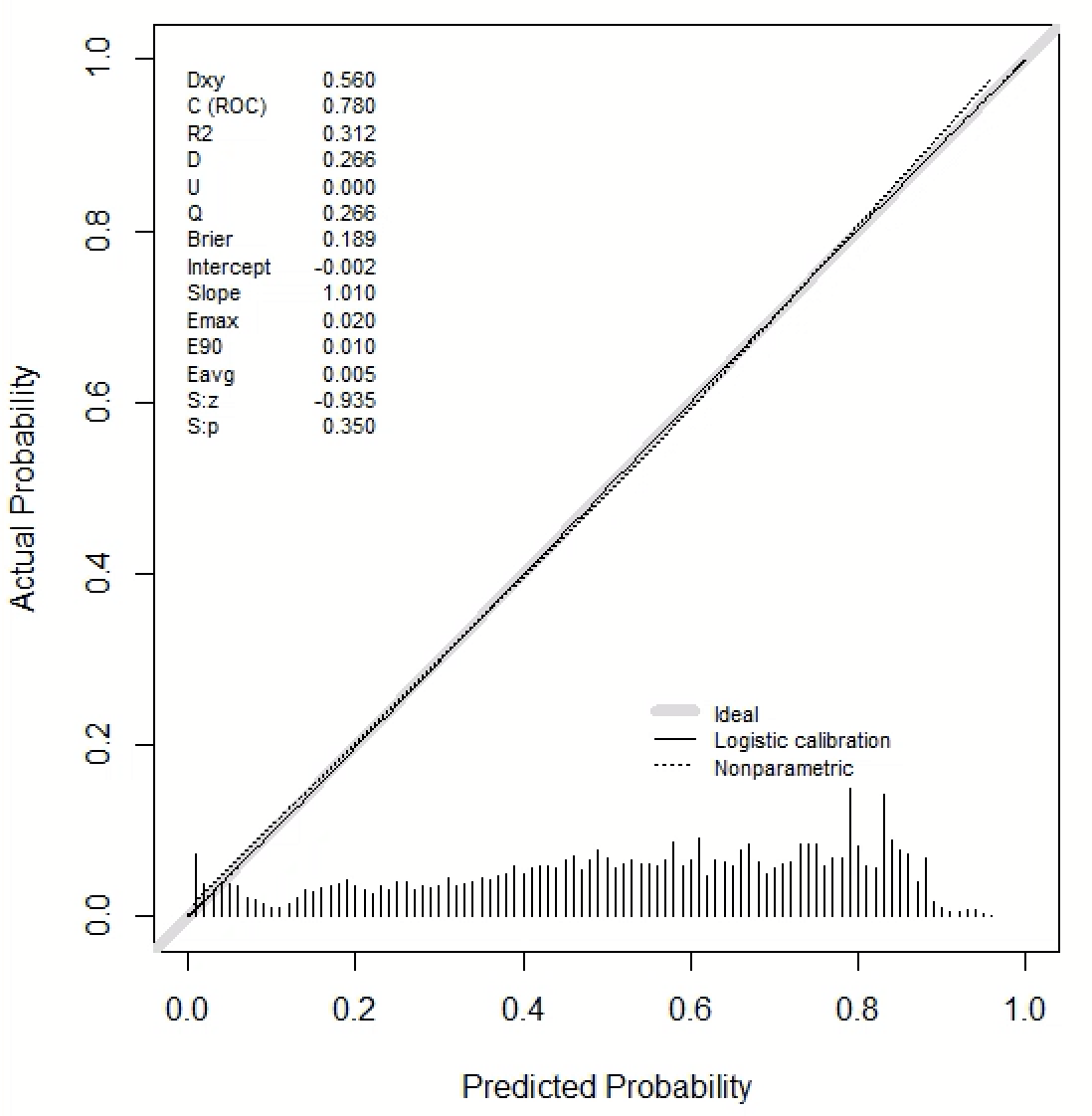

Supplement: S4 Fig — (TIF) [file pone.0289429.s005.tif]
