# Supplementary material for: Prehospital prediction of hospital admission for emergent acuity patients transported by paramedics: A population-based cohort study using machine learning
Source: PLoS One. 2023 Aug 24;18(8):e0289429. doi: 10.1371/journal.pone.0289429 (PMC10449470; doi:10.1371/journal.pone.0289429)
Supplement: S2 Table — (DOCX) [file pone.0289429.s007.docx]

**S2 Table. Hyperparameter tuning details.**

| **Machine Learning Algorithm** | **Details of Hyperparameter Tuning, Optimal Parameters and Predictive Accuracy** | | |
| --- | --- | --- | --- |
| **Random Forest** | **Parameter** | Number of Splitting Variables (mtry) | Minimum Node Size |
|  | **Range** | 2 – 10 | 1, 5, 10, 15 |
|  | **Optimal** | 5 | 15 |
| **Gradient Boosted Trees** | **Parameter** | Maximum Tree Depth | Gamma |
|  | **Range** | 2 – 8 | 0.0001 – 1 |
|  | **Optimal** | 5 | 0.0004 |
|  | **Parameter** | Maximum Boosting Iterations | Learning Rate (ETA) |
|  | **Range** | 50 – 5,000 | 0.001 to 0.3 |
|  | **Optimal** | 418 | 0.0345 |
|  | **Parameter** | Subsample Ratio; Columns | Subsample Ratio; Training |
|  | **Range** | 0.25 – 0.9 | 0.5 – 0.9 |
|  | **Optimal** | 0.561 | 0.601 |
| **Logistic Regression** | Not Applicable. | | |
| **Lasso Logistic Regression** | Not Applicable. | | |
