# Supplementary material for: Prehospital prediction of hospital admission for emergent acuity patients transported by paramedics: A population-based cohort study using machine learning
Source: PLoS One. 2023 Aug 24;18(8):e0289429. doi: 10.1371/journal.pone.0289429 (PMC10449470; doi:10.1371/journal.pone.0289429)
Supplement: S3 Table — Results of variable importance predictors for each machine learning algorithm. (DOCX) [file pone.0289429.s008.docx]

**S3 Table. Variable Importance.** Results of variable importance predictors for each machine learning algorithm.

| **Variable (Patient Characteristic)** | **LR** | **Lasso LR** | **RF** | **GBT** |
| --- | --- | --- | --- | --- |
| **Age Group**  18-39  40-64  65-105 | (ref)  -0.014  -0.087 | (ref)  -0.011  -0.077 | (ref)  -0.012  -0.054 | (ref)  -0.006  -0.045 |
| **Sex**  Male  Female | -0.001  (ref) | -0.001  (ref) | -0.010  (ref) | -0.005  (ref) |
| **Access to Primary Health care*** | -0.000 | -0.000 | -0.006 | -0.002 |
| **Comorbidities***  Hypertension  Diabetes  COPD  Asthma  Rheumatoid Arthritis  CHF  Bowel Disease-  Cancer | -0.002  -0.002  -0.000  -0.001  -0.000  -0.003  -0.000  -0.002 | -0.002  -0.001  -0.000  -0.001  -0.000  -0.002  -0.000  -0.002 | -0.013  -0.012  -0.010  -0.006  -0.002  -0.010  -0.001  -0.010 | -0.003  -0.004  -0.004  -0.001  -0.001  -0.003  -0.001  -0.005 |
| **Presenting Complaint**  Cardiac Arrest  Cardiovascular  ENT  Environmental  Gastrointestinal  Genitourinary  Mental Health  Neurologic  Obstetrician-Gynecological  Ophthalmology  Orthopedic  Other  Respiratory  Skin  Substance Misuse  Trauma  General and Minor | (ref)  -0.000  -0.002  -0.000  -0.012  -0.000  -0.008  -0.006  -0.001  -0.001  -0.001  -0.001  -0.042  -0.000  -0.000  -0.000  -0.017 | (ref)  -0.010  -0.004  -0.000  -0.002  -0.001  -0.002  -0.000  -0.000  -0.001  -0.000  -0.000  -0.021  -0.000  -0.001  -0.000  -0.008 | (ref)  -0.015  -0.003  -0.000  -0.006  -0.002  -0.005  -0.003  -0.000  -0.000  -0.002  -0.000  -0.021  -0.000  -0.003  -0.001  -0.009 | (ref)  -0.011  -0.002  -0.000  -0.014  -0.000  -0.006  -0.008  -0.000  -0.001  -0.008  -0.000  -0.042  -0.000  -0.001  -0.002  -0.016 |
| **Geographic Location**  Urban  Rural | -0.001  (ref) | -0.000  (ref) | -0.002  (ref) | -0.000  (ref) |
| **Referral Source**  Self/Family Member  Ambulatory Care Services  Private Practice  Residential Care Facility  Other | -0.002  -0.000  -0.000  -0.037  (ref) | -0.002  -0.000  -0.000  -0.037  (ref) | -0.009  -0.001  -0.001  -0.026  (ref) | -0.005  -0.000  -0.000  -0.036  (ref) |
| **Receiving Home Care*** | -0.013 | -0.014 | -0.031 | -0.017 |
| **Note:** shown as the reduction in area under the receiving operator curve in absence of predictor. LR = logistic regression, Lasso LR = lasso logistic regression, RF = random forest, GBT = gradient boosted trees.  * ‘No’ is reference group. | | | | |
