# Supplementary material for: Prehospital prediction of hospital admission for emergent acuity patients transported by paramedics: A population-based cohort study using machine learning
Source: PLoS One. 2023 Aug 24;18(8):e0289429. doi: 10.1371/journal.pone.0289429 (PMC10449470; doi:10.1371/journal.pone.0289429)
Supplement: S4 Table — Variable-relevance ranking of top 10 predictors for each machine learning algorithm. (DOCX) [file pone.0289429.s009.docx]

**S4 Table. Variable-relevance ranking.** Variable-relevance ranking of top 10 predictors for each machine learning algorithm.

| **Rank** | **LR** | **Lasso LR** | **RF** | **GBT** |
| --- | --- | --- | --- | --- |
| 1 | Age group 65-105 years | Age group 65-105 years | Age group 65-105 years | Age group 65-105 years |
| 2 | Presenting complaint is respiratory | Referral source from a residential care facility | Receiving home care | Presenting complaint is respiratory |
| 3 | Referral source from a residential care facility | Presenting complaint is respiratory | Referral source from a residential care facility | Referral source from a residential care facility |
| 4 | Presenting complaint is general or minor | Receiving home care | Presenting complaint is respiratory | Receiving home care |
| 5 | Age group 40-64 years | Age group 40-64 years | Presenting complaint is cardiovascular | Presenting complaint is general or minor |
| 6 | Receiving home care | Presenting complaint is cardiovascular | Comorbidity of hypertension | Presenting complaint is gastrointestinal |
| 7 | Presenting complaint is gastrointestinal | Presenting complaint is general or minor | Age group 40-64 years | Presenting complaint is cardiovascular |
| 8 | Presenting complaint is mental health | Presenting complaint is ears, nose or throat | Comorbidity of diabetes | Presenting complaint is neurological |
| 9 | Presenting complaint is neurological | Comorbidity of hypertension | Sex is male | Presenting complaint is orthopedic |
| 10 | Comorbidity of congestive heart failure | Comorbidity of congestive heart failure | Comorbidity of chronic obstructive pulmonary disease | Age group 40-64 years |
| LR = logistic regression, Lasso LR = lasso logistic regression, RF = random forest, GBT = gradient boosted trees. | | | | |
